# Genetic Interaction between Mutations in c-Myb and the KIX Domains of CBP and p300 Affects Multiple Blood Cell Lineages and Influences Both Gene Activation and Repression

**DOI:** 10.1371/journal.pone.0082684

**Published:** 2013-12-10

**Authors:** Lawryn H. Kasper, Tomofusa Fukuyama, Stephanie Lerach, Yunchao Chang, Wu Xu, Song Wu, Kelli L. Boyd, Paul K. Brindle

**Affiliations:** 1 Department of Biochemistry, St. Jude Children’s Research Hospital, Memphis, Tennessee, United States of America; 2 Department of Biostatistics, St. Jude Children’s Research Hospital, Memphis, Tennessee, United States of America; 3 Veterinary Pathology Core, St. Jude Children’s Research Hospital, Memphis, Tennessee, United States of America; Cincinnati Children's Hospital Medical Center, United States of America

## Abstract

Adult blood cell production or definitive hematopoiesis requires the transcription factor c-Myb. The closely related KAT3 histone acetyltransferases CBP (CREBBP) and p300 (EP300) bind c-Myb through their KIX domains and mice homozygous for a p300 KIX domain mutation exhibit multiple blood defects. Perplexingly, mice homozygous for the same KIX domain mutation in CBP have normal blood. Here we test the hypothesis that the CBP KIX domain contributes subordinately to hematopoiesis via a genetic interaction with c-Myb. We assessed hematopoiesis in mice bearing compound mutations of c-Myb and/or the KIX domains of CBP and p300, and measured the effect of KIX domain mutations on c-Myb-dependent gene expression. We found that in the context of a p300 KIX mutation, the CBP KIX domain mutation affects platelets, B cells, T cells, and red cells. Gene interaction (epistasis) analysis provides mechanistic evidence that blood defects in KIX mutant mice are consistent with reduced c-Myb and KIX interaction. Lastly, we demonstrated that the CBP and p300 KIX domains contribute to both c-Myb-dependent gene activation and repression. Together these results suggest that the KIX domains of CBP, and especially p300, are principal mediators of c-Myb-dependent gene activation and repression that is required for definitive hematopoiesis.

## Introduction

The development of all adult hematopoietic stem cell-derived blood cell lineages requires the transcription factor c-Myb, although how it controls hematopoiesis remains unclear [1,2]. Transcription factors regulate target gene activity through interactions with coactivators and corepressors, and the BIOGRID database (thebiogrid.org, [3]) reports more than fifty c-Myb interacting proteins in mice and humans [4-47]. Although generally thought of as an activator of gene expression, recent findings show that c-Myb can also directly repress target genes [48]; however, details of how c-Myb-interaction partners affect these opposite transcriptional effects, and hematopoiesis, are not well understood.

Perhaps the most widely studied of c-Myb partners, CBP (CREB binding protein, Crebbp) and p300 (E1A binding protein p300, Ep300) reportedly interact with more than 400 other proteins [49]. Together CBP and p300 form the KAT3 family of acetyltransferases that add acetyl groups to lysines in histones and other proteins, although which of these CBP/p300 substrates are critical for gene regulation remains uncertain [50]. Histone acetylation is often associated with gene activation [51] and CBP and p300 are usually described as coactivators of gene expression; however, paradoxically p300 has been implicated in both direct activation and repression of genes by c-Myb [48].

**Figure 1:**
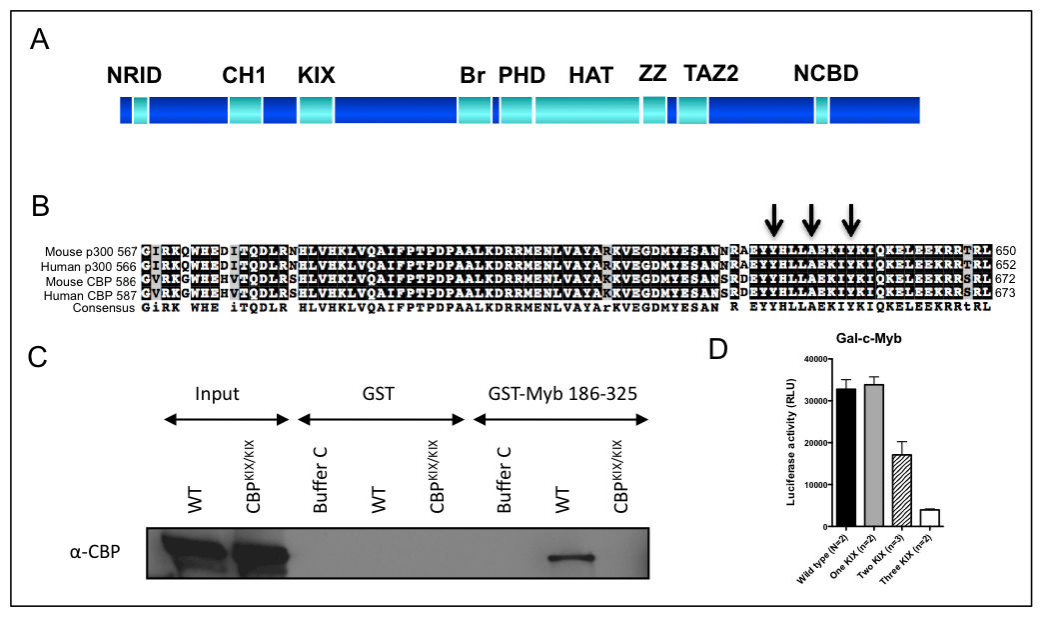
The KIX domains of both CBP and p300 contribute to c-Myb transactivation function in a dose dependent manner. (A) Schematic showing approximate locations of CBP and p300 domains [91]. Nuclear receptor interaction domain (NRID), the CREB-binding domain (KIX), Cys/His-rich region-1 (CH1 [57] or transcriptional-adaptor zinc-finger-1, TAZ1), bromodomain (Br), plant homeodomain (PHD), HAT enzymatic region, zinc-binding domain (ZZ), TAZ2 (aka CH3 [92]), and the nuclear receptor binding domain (NCBD). (B) Alignment of the KIX domains of mouse and human p300 and CBP. Amino acids mutated in the KIX domain mutants are indicated with arrows. (C) Pulldown assay with the c-Myb transactivation domain (aa186-325) fused to GST using extracts from wild type (WT) and *CBP* ^KIX/KIX^ mouse embryonic fibroblasts. Buffer C is extraction buffer without protein. (D) Transient transfection assay showing Gal-c-Myb 186-325 transactivation function is reduced in mouse embryonic fibroblasts having the indicated number of *CBP* and p300 mutant KIX alleles (one (*p300^+/KIX^* or *CBP^+/KIX^*), two (*CBP^KIX/KIX^, p300^KIX/KIX^* or *p300^+/KIX^*;*CBP^+/KIX^*) or three (*p300^+/KIX^*;*CBP^KIX/KIX^* or *p300^KIX/KIX^*;*CBP* ^+/KIX^) KIX); wild type MEFs indicated; one MEF isolate of each genotype except wild type (N=2); (mean ± SEM).

Normal human development requires CBP and p300 [52,53], and mice lacking either CBP or p300 die before birth [54,55]. This lethality has necessitated studies using either conditional knockout alleles, which allow tissue specific gene inactivation or “knock-in” mice in which CBP and p300 proteins are expressed at normal levels but only specific protein interaction domains are mutated (e.g. CH 1, KIX, Figure 1A) [56-58]. The use of domain-specific knock-ins is especially useful in limiting the effect of CBP and p300 mutations to a few specific CBP/p300-interacting proteins out of the hundreds that have been reported [49]. 

We previously designed a triple point mutation (Y631A, A635Q and Y639A) in the KIX domain of p300 that alters the binding surface for c-Myb and CREB, but which should maintain the secondary and tertiary structure of the domain (Figure 1B)[56]. In this way, these point mutations should specifically block proteins from binding that particular surface of the KIX domain, and not interfere with other surfaces of KIX or other domains of CBP/p300 and their protein interactions. To date, only CREB and c-Myb interactions have been shown to be affected by this mutation [56,59]. We showed previously that mice homozygous for this triple point mutation in the p300 KIX domain exhibit multi-lineage defects in hematopoiesis including severe anemia, B and T cell deficiencies, abnormal megakaryocytes and elevated platelet counts (Table 1) [56]. While blood from mice heterozygous for either a *c-Myb* null allele or the *p300* KIX triple point mutation appears normal, combining these mutations in the same mouse (*p300* ^*+/KIX*^;*c-Myb* ^*+/-*^) synergistically affects megakaryocyte development and increases platelet counts without obvious changes in other peripheral blood cell numbers (Table 1) [56]. This gene interaction (epistasis) experiment highlights the critical nature of the genetic interaction between c-Myb and the p300 KIX domain for normal megakaryocyte development *in vivo*. It remains unclear, however, whether other blood cell lineages also require the genetic interaction of c-Myb with the p300 or CBP KIX domain. 

**Table 1:** Comparison of blood cell phenotypes between different c-Myb and p300 and/or CBP KIX domain mutant mice.

Relative blood cell abundance	Red cells	megs/ platelets	B cells	T cells	thymo-cytes	mono-cytes	neutro-phils	refs.
c-Myb^-/-^	none	none**^*a*^**	none	none	none	none	none	[1,82]
c-Myb^-/flox b^	low	v high	v low	no info	v low	high	v low	[83]
c-Myb ^Plt4/Plt4 c^	low	v high	low**^*d*^**	low**^*d*^**	no info	high	high	[90]
c-Myb^M303V/M303V^	low	v high	v low	low	low	norm	norm	[25]
p300^Plt6/Plt6 e^	norm	high	low	norm	no info	norm	norm	[81]
p300^KIX/KIX f^	v low	v high	v low	norm	low	norm	norm	[56]
p300^+/KIX^; c-Myb^+/- f^	norm	high	norm	norm	low	norm	norm	[56]
p300^+/KIX^; CBP^KIX/KIX f^	low	v high	low	low	v low	norm	norm	here
p300^+/KIX^; CBP^+/KIX^;c-Myb^+/- f^	low	v high	v low	low	low	norm	norm	here
p300^+/KIX^	norm	norm	norm	norm	n.d.	norm	norm	here
p300^+/KIX^; CBP^+/KIX^	norm	high	norm	norm	n.d.	norm	norm	here
c-Myb^+/-^	norm	norm	low	norm	n.d.	norm	norm	here

Blood cell numbers are estimated compared to controls within own study as absent (none), very low (v low), low, normal (norm), high or very high (v high). (no info) is indicated where no numbers for a cell type were reported in the original study and n.d. indicates analysis was not done. **^*a*^**Original c-Myb knockout mice indicated that megakaryocytic lineage was spared to some degree [1], but follow up work using chimeric mice showed that c-Myb-/- cells did not contribute to megakaryocytes in adult mice [82]. **^*b*^**The c-Myb flox allele is a hypomorph resulting in a reduced c-Myb protein level. c-Myb**^*-*^** ^/flox^ mice have 5-10% of normal c-Myb protein expression [83]. **^*c*^**The ENU induced Plt4 mutation equates to c-Myb V384D [90]. **^*d*^**Total lymphocyte count from peripheral blood given, but not split out into B and T cells [90]. **^*e*^**The ENU induced Plt6 mutation equates to p300 Y630N in the KIX domain [81]. **^*f*^**The KIX mutation is a triple point mutation: Y631A, A635Q and Y639A in p300 or Y650A, A654Q and Y658A in CBP.

Although the sequence of the KIX domain of CBP is 90% identical to the p300 KIX domain, hematopoiesis in mice homozygous for a comparable mutation in the KIX domain of CBP (Y650A, A654Q and Y658A, Figure 1B) appeared essentially normal [56]. This disparity may be due to differences in protein expression (i.e. CBP and p300 have the same functions in blood cells) or biochemical properties (i.e. p300 has a different and more critical function in blood cells than CBP). While the KIX domain of p300 seems to be more critical in hematopoiesis than the KIX domain of CBP, multiple studies have established that both holo-CBP and -p300 have important roles in normal blood cell production. Some of these roles seem to be unique to either CBP or p300; for example, analyses of aged mice revealed an increased prevalence of hematopoietic tumors and abnormalities affecting the B cell and myeloid lineages in mice heterozygous for a null allele of CBP, although no such defects were found in p300 null heterozygotes [60]. Similarly, CBP null, but not p300 null hematopoietic stem cells lose self renewal capacity, while p300 null, but not CBP null embryonic stem cells fail in normal blood cell differentiation *in vitro* [61]. Lineage-specific conditional knockout of CBP and p300 in specific lymphoid compartments have demonstrated that while CBP and p300 are each modestly important for B cells [62], CBP has a unique role in demarcating the development of innate and conventional T cells [63,64]. Importantly, Kimbrel et al. showed that three copies of *CBP*, the two endogenous alleles plus a *CBP* cDNA expressed from the *p300* promoter, could rescue hematopoiesis in the absence of p300 protein [65]. This suggests that protein expression levels, rather than biochemical properties, may account for the distinct effects of mutating the KIX domains of CBP and p300 on hematopoiesis.

In this study we sought to answer four questions: 1) Does the KIX domain of CBP have a role in hematopoiesis? 2) Is genetic interaction between c-Myb and CBP/p300 KIX necessary for the normal production of other blood cell lineages besides megakaryocytes? 3) Does genetic interaction between c-Myb and KIX largely explain the phenotype of c-Myb and KIX mutant mice? and 4) Is the genetic interaction between c-Myb and KIX necessary for both activation and repression of c-Myb-dependent genes? To answer these questions, we assessed blood cell production in mice having compound mutations in c-Myb and/or the KIX domains of CBP and p300 and quantified the effect of KIX domain mutations on c-Myb-dependent changes in gene expression. Our findings suggest that through their KIX domains, CBP, and particularly p300, are the main effectors of the c-Myb-dependent gene activation and repression required for normal hematopoiesis.

## Material and Methods

### Animals

Animal experiments were approved by the St Jude Institutional Animal Care and Use Committee and performed in accordance with IACUC guidelines. Generation of the *p300* and *CBP* KIX mutant alleles (Mouse Genome Informatics IDs 3578128 and 3578129) was described in Kasper et al. [56]. Note that the numbering of the p300 KIX mutations Tyr631Ala, Ala635Gln, and Tyr639Ala now coincide with more recent mouse p300 protein sequences (NCBI NP_808489.4 and Ensembl ENSMUSP00000066789) and are one position different than those originally listed in 2002. The *c-Myb* knockout allele (MGI ID 2662859) was described in Mucenski et al. [1]. Unless otherwise noted, mice analyzed in this study were C57BL/6J x 129Sv F1 hybrids generated from multiply-backcrossed congenic lines.

### Plasmids and transient transfection assays

The Gal-c-Myb plasmid (aa 186 to 325) was described in Parker et al. [66]. The GST-c-Myb 186-325 plasmid was constructed in pGEX 4T-3 (GE Life Sciences) using an EcoRI/SmaI fragment from Gal-c-Myb containing the c-Myb aa186 to 325 insert. Transient transfection assays using MEFs were performed as previously described using the Promega Dual Luciferase Reporter Assay Kit where reporter gene luciferase activity is normalized to cotransfected *Renilla* luciferase reporter activity [59]. MSCV c-Myb IRES GFP was a gift of Angelika Hoffmeyer.

### Flow cytometry, FACS and hematopoietic analysis

Flow cytometry was performed on BD Biosciences FACS Calibur and FACS LSR instruments. FACS of CD4^+^CD8^+^ double positive thymocytes was performed on a BD Biosciences Aria. All antibodies were from Becton Dickenson, except the IL18R antibody was from R&D Systems. Complete blood counts were generated using a Hemavet Hematology System (Drew Scientific). Hematocrits were determined manually. 

### Statistical method for calculating genetic interaction (epistasis)

As defined by Jasnos et al., epistasis (*E*) was considered absent if the multiplicative fitness phenotypes of the extreme genotypes equaled that of the intermediate genotypes [67]; i.e. (WT0 * TH3) = (CM1*CP2), where WT0 = wild type phenotype, CM1= c-Myb^+/-^, CP2 = *CBP* ^+/KIX^;*p300* ^*+/KIX*^, and TH3 = c-Myb^+/-^;*CBP* ^*+/KIX*^;*p300* ^*+/KIX*^. Blood cell counts were log transformed to stabilize their variance, which coverts the formula to *E* = (WT0 + TH3) – (CM1 + CP2). There is no epistasis if *E* = 0, whereas *E* > 0 defines positive epistasis (higher than expected phenotype), and *E* < 0 signifies negative epistasis (lower or worse than expected phenotype) [68]. Because the machine-derived cell count data were obtained from independent experiments performed on different dates, the experiments were treated as a random effect in our model to account for machine drift over time. Thus, a mixed model was used (SAS 9.1.3 software). Since no significant strain effect was detected between the C57BL/6J and C57BL/6J x 129Sv F1 mice used in this analysis, their data were combined to increase the power of the analysis. Mice used were one to two months old; the effect of age in this cohort was not significant and is excluded from the model. The mean and 95% confidence intervals were calculated for each cell type tested, and no multiple comparison adjustment was performed for this analysis.

### Mouse embryonic fibroblasts (MEFs) and retroviral infection

Primary MEFs were generated as described from e14.5 C57BL/6J x 129Sv F1 embryos and were used between passage three and six [69]. Retrovirus was generated from MSCV-IRES-GFP (GFP control virus) and MSCV-c-Myb-IRES-GFP plasmids. MEFs were infected with retrovirus 6 days before RNA harvest.

### Gene expression

RNA was isolated using Trizol (Life Technologies). Microarray platforms used were Affymetrix Mouse Genome 430v2 Arrays (MEF experiment) and Affymetrix HT MG-430 PM Arrays (KIX CD4^+^CD8^+^ double positive thymocyte experiment) and were analyzed using Spotfire software (TIBCO). Array data were deposited with ArrayExpress (E-MTAB-1973 and E-MTAB-1974). Reverse transcriptase reactions were performed using Superscript II (Life Technologies). qPCR was performed on MJ Research Opticon and BioRad CFX Connect real time machines using Quantitect SYBR Master Mix (Qiagen). Gene expression datasets from T cell specific inactivation of c-Myb in double positive thymocytes (*c-Myb* ^*flox/flox*^;CD4-Cre;*Tcrα* ^*-/-*^ mice compared with *c-Myb* ^*+/flox*^;CD4-Cre;*Tcrα* ^*-/-*^) are from Yuan et al. [70]. Gene set derived from analysis of wild type and *c-Myb* ^Plt4/Plt4^ Lin^-^ Sca-1^+^ c-kit^+^ (LSK) hematopoietic precursor cells was from Greig et al. [71]. 

## Results

### Both CBP and p300 KIX contribute to c-Myb function *in vitro*

Although the KIX domain of CBP can directly bind the c-Myb transactivation domain *in vitro*, and the interaction is sensitive to our KIX mutation (Figure 1C, [20,56]), only mutation of the p300 KIX domain has previously been shown to affect hematopoiesis *in vivo* [56]. We also showed that c-Myb transactivation function in both *p300* ^*KIX/KIX*^ and *CBP* ^KIX/KIX^ mouse embryonic fibroblasts (MEFs) is reduced, demonstrating that both CBP and p300 contribute to c-Myb activity in an *in vitro* assay [56]. While a single mutant KIX allele of either CBP or p300 produced no effect on transactivation by a Gal-c-Myb construct, we found that increasing the number of mutant KIX alleles of CBP and p300 in MEFs from two (*CBP* ^KIX/KIX^ *, p300* ^*KIX/KIX*^ or *p300* ^*+/KIX*^;*CBP* ^+/KIX^) to three (*p300* ^*+/KIX*^;*CBP* ^KIX/KIX^ or *p300* ^*KIX/KIX*^;*CBP* ^+/KIX^) further decreased the activity (Figure 1D). This led us to predict that an additional KIX mutant allele of CBP or p300 would worsen the *in vivo* c-Myb-dependent phenotype of a *p300* ^*KIX/KIX*^ or *CBP* ^KIX/KIX^ mouse, and could reveal a role for the CBP KIX domain in hematopoiesis. To this end, we generated mice bearing mutations in both p300 KIX and CBP KIX and examined the effect on blood cell production.

### Like c-*Myb* mutants, *p300* ^*KIX/KIX*^;*CBP* ^+/KIX^ and *p300* ^*+/KIX*^;*CBP* ^KIX/KIX^ embryos are grossly normal but display defective blood cell formation

**Figure 2:**
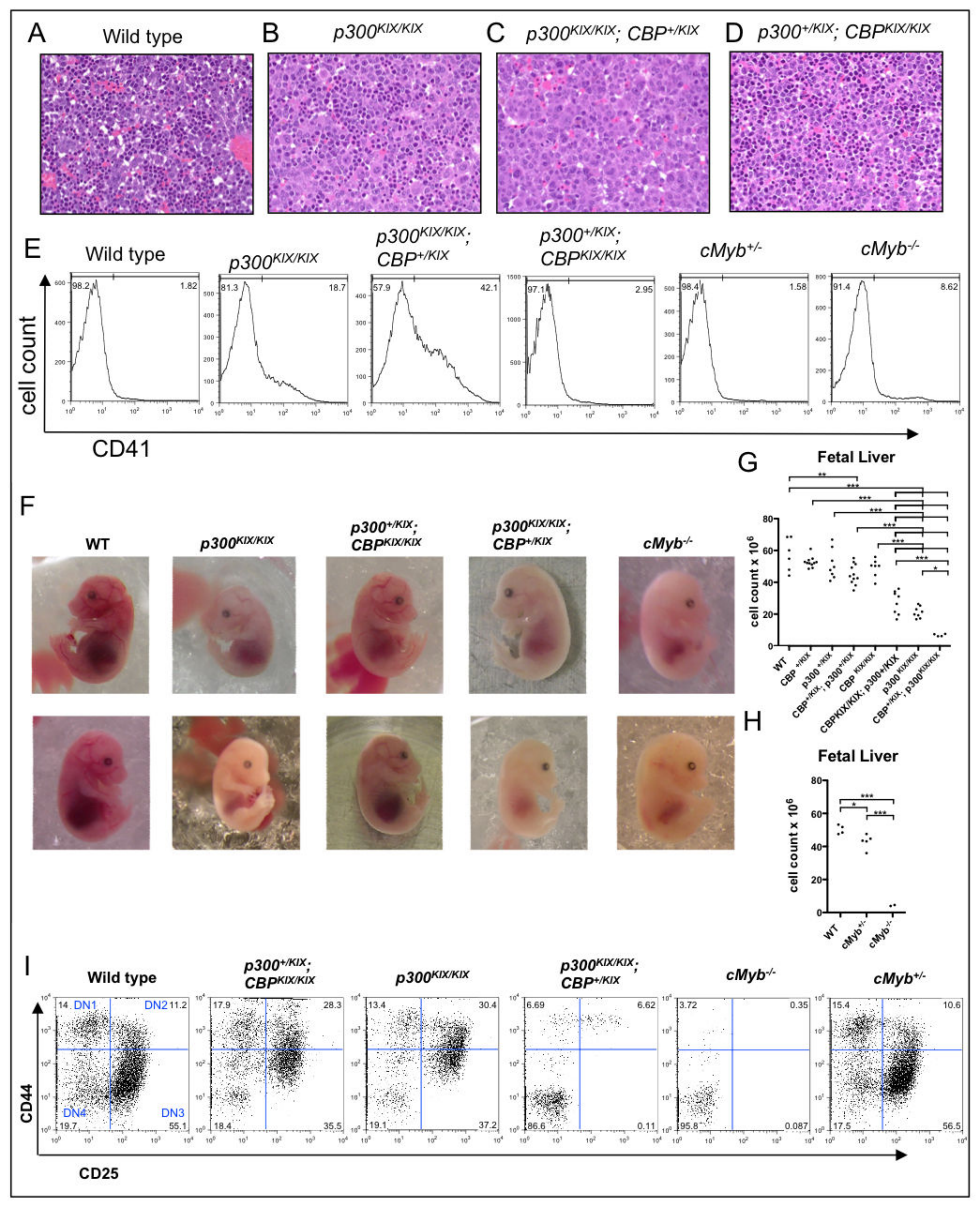
The KIX domains of CBP and p300 contribute to adult blood cell production in the fetus, albeit nonequivalently. (**A**-**D**) e15.5 fetal liver sections, H & E staining, 400x magnification. (**E**) Flow cytometry on e15.5 fetal liver cells showing an increased CD41^+^ megakaryocyte population *in* p300^*KIX/KIX*^ and p300^*KIX/KIX*^;*CBP* ^+/KIX^ mice. (**F**) e15.5 embryos demonstrating anemia-associated pallor *in* p300^*KIX/KIX*^, p300^*KIX/KIX*^;*CBP* ^+/KIX^ and c-Myb^-/-^ embryos. (**G**,**H**) Cell counts for e15.5 fetal livers. For both the KIX and Myb mutant panels, the analysis was a one-way ANOVA (p<0.0001) with Tukey post test between genotype pairs. Non significant pairings are not marked and significant pairings are indicated * p<0.05, ** p<0.01 and *** p<0.001. For the KIX panel, N=4-11 for each genotype, for the Myb panel N=2 (Myb KO) to 5. (**I**) CD25/CD44 flow cytometry on e15.5 fetal thymocytes gated first for CD4^-^CD8^-^ (double negative, DN) subset. Embryos are C57BL/6J x 129Sv F2 background except for c-Myb^+/-^ and c-Myb^-/-^ embryos, which were C57BL/6J. DN stages shown in wild type.

While yolk sac-derived primitive (embryonic) hematopoiesis is c-Myb-independent [2], c-Myb-dependent definitive (adult) hematopoiesis initially occurs in the fetal liver. We first looked at the impact of combining p300 KIX and CBP KIX mutant alleles in e15.5 fetal liver, since defective hematopoiesis, including decreased red cell production and increased numbers of megakaryocytes (the cell type that produces platelets) is evident at this stage in *p300* ^*KIX/KIX*^ embryos (Figure 2A, B, E and G). We found that adding one CBP KIX mutant allele (*p300* ^*KIX/KIX*^;*CBP* ^+/KIX^) produced embryos that were grossly normal but had pale fetal livers (Figure 2F) with reduced cell counts (Figure 2G) and dramatically reduced red cell production rendering adult survival unlikely (Figure 2C). The *p300* ^*KIX/KIX*^;*CBP* ^+/KIX^ fetal livers had a pallor similar in appearance to that of *c-Myb* ^*-/-*^ embryos (Figure 2F), and cell counts in *p300* ^*KIX/KIX*^;*CBP* ^+/KIX^ and *c-Myb* ^*-/-*^ fetal livers were similarly deficient (Figure 2G and H), suggesting that the major effect of simultaneously mutating the p300 and CBP KIX domains is on c-Myb function. At the cellular level, *p300* ^*KIX/KIX*^;*CBP* ^+/KIX^ fetal livers also contained even more cells positive for the megakaryocyte marker CD41 than *p300* ^*KIX/KIX*^ fetal livers (Figure 2E). By contrast, embryos with one wild type p300 allele (*p300* ^*+/KIX*^;*CBP* ^KIX/KIX^) appeared to have only a mild deficit in fetal liver erythropoiesis (Figure 2D, 2F), and their livers contained normal numbers of megakaryocytes (Figure 2E). 

We next looked at developmental stages within the CD4^-^CD8^-^ double negative (DN) population of e15.5 thymuses (at e15.5, fetal thymuses contain primarily CD4^-^CD8^-^ cells [72]) by staining for expression of the developmental markers CD44 and CD25 (stages from earliest to latest are: CD44^+^CD25^-^ (DN1), CD44^+^CD25^+^ (DN2), CD44^-^CD25^+^ (DN3) and CD44^-^CD25^-^ (DN4)). This analysis showed that both *p300* ^*KIX/KIX*^ and *p300* ^*+/KIX*^;*CBP* ^KIX/KIX^ embryos exhibit a partial block in progression from DN2 to DN3 with the DN2/DN3 ratio being close to 1:1 compared with the 1:5 ratio seen in wild type and *c-Myb* ^*+/-*^ controls (Figure 2I). In contrast, *p300* ^*KIX/KIX*^;*CBP* ^+/KIX^ and *c-Myb* ^*-/-*^ CD4^-^CD8^-^ double negative cells are almost entirely CD44^-^CD25^-^ (>85% compared with 15-20% in controls; Figure 2I). The most likely explanation for this unusual CD44/CD25 profile is that most of the CD4^-^CD8^-^ double negative cells found in the *p300* ^*KIX/KIX*^;*CBP* ^+/KIX^ and *c-Myb* ^*-/-*^ fetal thymuses are stromal cells rather than DN4 stage thymocytes. Together, these data suggest that while the KIX domain of p300 has a more critical role, the KIX domain of CBP also contributes to definitive hematopoiesis in the mouse embryo. In addition, the milder phenotype of *p300* ^*+/KIX*^;*CBP* ^KIX/KIX^ embryos (Figure 2F) indicated that they survive to adulthood and could provide a model for examining the role of the CBP KIX domain in adult mouse hematopoiesis.

### Multiple blood cell lineages require the KIX domains of CBP and p300

**Figure 3:**
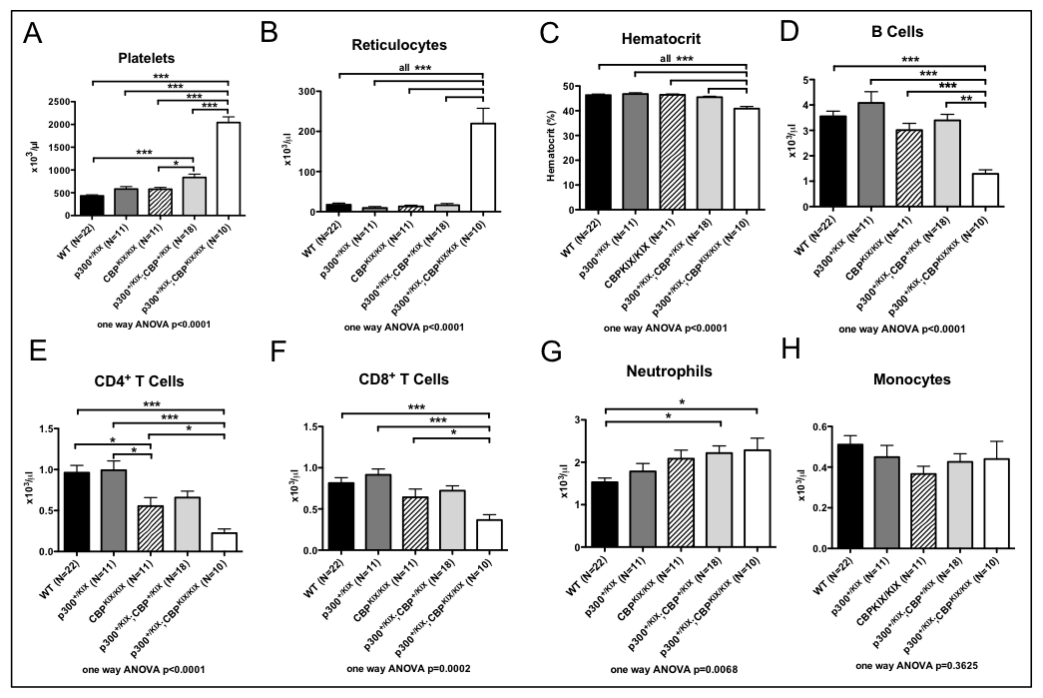
Triple-KIX p300^*+/KIX*^; *CBP* ^KIX/KIX^ adult mice have defective hematopoiesis affecting multiple lineages. Peripheral blood counts from 1-4 month old C57BL/6J x 129Sv F1 wild type (WT) and KIX mutant mice (N=10-22). Counts from automated Hemavet complete blood count. For B cells and T cells, total lymphocyte counts from complete blood count were parsed using flow cytometry to determine the percentage of lymphocytes that were positive for B220 (B cells), CD3 (T cells), CD4 and CD8 (T cell subsets). Asterisks indicate significant p value by pairwise Tukey post test following one way ANOVA (* p<0.05, ** p<0.01, *** p<0.001).

Only about 40% of *p300* ^*+/KIX*^;*CBP* ^KIX/KIX^ (triple-KIX) mice reached weaning age; however, the surviving mice appeared grossly normal (SLL, LHK data not shown). We examined the peripheral blood of 1-4 month old wild type (WT), triple-KIX *p300* ^*+/KIX*^;*CBP* ^KIX/KIX^ and intermediate genotype control mice (*p300* ^*+/KIX*^ single heterozygous, *CBP* ^KIX/KIX^ homozygous, and *p300* ^*+/KIX*^;*CBP* ^+/KIX^ compound heterozygous) by flow cytometry and complete blood count to determine the effect of mutant KIX alleles on cell numbers. We found that in triple-KIX *p300* ^*+/KIX*^;*CBP* ^KIX/KIX^ mice, both platelet (Figure 3A) and reticulocyte (immature red cell, Figure 3B) counts were significantly elevated above all control mice. By contrast, triple-KIX *p300* ^*+/KIX*^;*CBP* ^KIX/KIX^ mice displayed significantly lower hematocrits (percentage of red cells by volume, Figure 3C) and B cell numbers (Figure 3D) compared with all controls. Counts of CD4^+^ and CD8^+^ T cells in peripheral blood showed that these cell types were significantly diminished in triple-KIX *p300* ^*+/KIX*^;*CBP* ^KIX/KIX^ mice compared with controls except for *p300* ^*+/KIX*^;*CBP* ^+/KIX^ compound heterozygous mice, which showed the same trend, but did not achieve statistical significance (Figure 3E,F; CD4^+^ T cells were also moderately reduced in *CBP* ^KIX/KIX^ mice). We also measured two myeloid lineage cells; neutrophil numbers showed a modest increase in triple-KIX *p300* ^*+/KIX*^;*CBP* ^KIX/KIX^ mice compared with wild type, but not the other intermediate genotypes (Figure 3G), and monocyte numbers were not significantly affected (Figure 3H, *p*=0.36). These data confirm that in the context of a single KIX mutant allele of *p300*, the KIX domain of CBP is critical for the production of normal numbers of B and T cells, red cells and platelets.

### Combining KIX and c-Myb mutant alleles results in epistatic effects on multiple blood cell lineages consistent with KIX and c-Myb acting in the same pathway

We showed previously [56] using the genetic epistasis method of complex haploinsufficient interaction analysis [73] that mice compound heterozygous for *p300* ^*KIX*^ and a *c-Myb* null allele exhibit abnormal megakaryocytes, increased platelet counts and reduced thymocyte numbers. These blood defects are not seen in *p300* ^*+/KIX*^ or *c-Myb* ^*+/-*^ single heterozygotes [56]. Consistent with the physical binding of c-Myb to the KIX domain [20,66], this genetic experiment provided *in vivo* evidence that a reduced interaction between p300 KIX and c-Myb is responsible for these aspects of the blood defect in *p300* ^*KIX/KIX*^ mice (Table 1, *p300* ^*KIX/KIX*^ mice display both increased platelet counts and decreased thymocyte numbers compared with wild type littermates) [56]. However, it remained unclear from that experiment whether other blood cell defects seen in *p300* ^*KIX/KIX*^ mice, such as anemia and decreased B cell counts, depend on a genetic interaction between *c-Myb* and *p300*. Based on the phenotypic overlap between *p300* ^*+/KIX*^;*CBP* ^KIX/KIX^ triple-KIX, *p300* ^*KIX/KIX*^ and various *c-Myb* mutant mice (Table 1), we hypothesized that additional blood lineages require the interaction of the KIX domain and c-Myb, and that *CBP*, as well as *p300*, participates in a genetic interaction with *c-Myb*.

**Figure 4:**
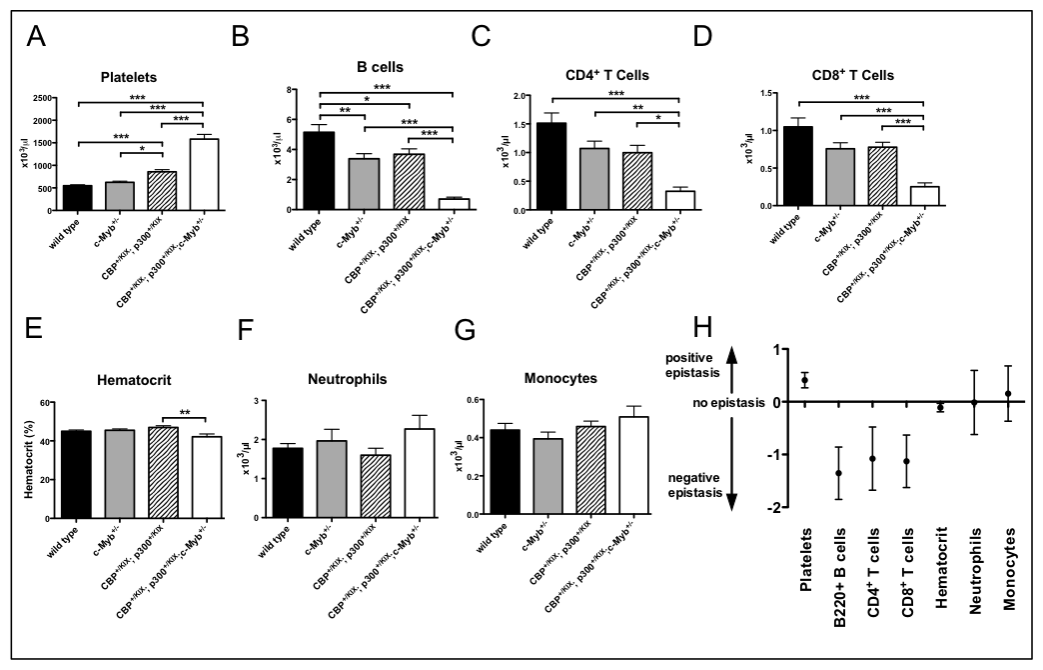
Combined KIX domain and c-Myb haploinsufficiency synergistically affects multiple blood lineages. Peripheral blood counts from 1-3 month old C57BL/6J x 129Sv F1 and C57BL/6J background mice. (A-G) Counts from automated Hemavet complete blood count. For B cells and T cells, total lymphocyte counts from complete blood count were parsed using flow cytometry to determine the percentage of lymphocytes that were positive for B220 (B cells), CD3 (T cells), CD4 and CD8 (T cell subsets). Asterisks indicate significant p value by pairwise Tukey post test following one way ANOVA (N=13-19 per genotype, * p<0.05, ** p<0.01, *** p<0.001). (H) Epistasis values and 95% confidence intervals calculated using five independent experiments with wild type, c-Myb^+/-^, p300^*+/KIX*^;*CBP* ^+/KIX^ and triple-het *p300^+/KIX^*;*CBP^+/KIX^*;c-Myb^+/-^ mice represented in each. Zero indicates no epistasis or the lack of synergistic interaction between mutations [i.e. equality of the multiplicative phenotype (or additivity of log-transformed phenotype in this case) of the extreme (wild type and *p300^+/KIX^*;*CBP^+/KIX^*;*c-Myb^+/-^*) and intermediate genotypes (*c-Myb^+/-^* and *p300^+/KIX^*;*CBP* ^+/KIX^)]. A positive epistasis value indicates a greater than multiplicative increased phenotype for the extreme genotype, a negative epistasis value indicates a greater than multiplicative decreased (aggravated) phenotype for the extreme genotype.

To test these hypotheses, we generated triple heterozygous (triple-het) *p300* ^*+/KIX*^;*CBP* ^*+/KIX*^;*c-Myb* ^*+/-*^ mice along with wild type, *p300* ^*+/KIX*^;*CBP* ^+/KIX^ and *c-Myb* ^*+/-*^ controls and analyzed their blood by flow cytometry and complete blood count. We found that the numbers of platelets, B cells and both CD4^+^ and CD8^+^ T cells differed significantly between triple-het *p300* ^*+/KIX*^;*CBP* ^*+/KIX*^;*c-Myb* ^*+/-*^ and all controls (Figure 4A-D). Triple-het *p300* ^*+/KIX*^;*CBP* ^*+/KIX*^;*c-Myb* ^*+/-*^ mice displayed a lower average hematocrit than controls (42% compared with 45-47%), but the measurement only achieved significance compared with *p300* ^*+/KIX*^;*CBP* ^+/KIX^ compound heterozygous mice (Figure 4E). Neither neutrophil nor monocyte numbers were significantly altered in triple-het *p300* ^*+/KIX*^;*CBP* ^*+/KIX*^;*c-Myb* ^*+/-*^ mice (Figure 4F and G). Although not tested here, it is possible that these myeloid cells are from a yolk-sac derived lineage that is known to be independent of c-Myb [74].

We next determined whether a complex haploinsufficiency interaction between *CBP* ^*KIX*^, *p300* ^*KIX*^ and *c-Myb* null mutant alleles existed by using a statistical model to calculate epistasis [67]. In this model, epistasis is present when the effect of combined mutations (in this case the triple-het *p300* ^*+/KIX*^;*CBP* ^*+/KIX*^;*c-Myb* ^*+/-*^) is greater than would be predicted from the phenotype of the intermediate genotypes (*p300* ^*+/KIX*^;*CBP* ^+/KIX^ and *c-Myb* ^*+/-*^). Positive epistasis (a higher than expected phenotype compared to intermediate controls) and negative epistasis (lower than expected phenotype) are represented by positive and negative values respectively; a value of zero indicates no epistasis [68]. Platelet counts in triple-het *p300* ^*+/KIX*^;*CBP* ^*+/KIX*^;*c-Myb* ^*+/-*^ mice showed the effect of positive epistasis (0.41 ± 0.15, mean epistasis value ± 95% confidence interval, Figure 4H), while B cell and both CD4^+^ and CD8^+^ T cell counts displayed negative epistasis (-1.35 ± 0.50, -1.08 ± 0.60 and -1.13 ± 0.50, Figure 4H). For hematocrit, the negative epistasis value was small; however, the 95% confidence interval did not overlap zero (-0.11 ± 0.08, Figure 4H). Neutrophils and monocytes in triple-het *p300* ^*+/KIX*^;*CBP* ^*+/KIX*^;*c-Myb* ^*+/-*^ mice demonstrated no epistatic effects (-0.013 ± 0.61 and 0.16 ± 0.52, Figure 4H). Overall, the analysis indicates that a genetic interaction between *c-Myb*, *CBP* and *p300* is limiting for the maintenance of normal numbers of platelets and lymphocytes. The impact of this triple mutation combination on hematocrit was less robust, but there is strong homeostatic pressure to maintain red cell production, so even a small variance in hematocrit may be significant. In fact, increased reticulocyte (immature red cell) counts seen in triple-het *p300* ^*+/KIX*^;*CBP* ^*+/KIX*^;*c-Myb* ^*+/-*^ mice show that red cell production is stressed and support the assertion that compensatory mechanisms are at work (Figure S1).

### Both c-Myb activated and repressed transcription requires the KIX domains of CBP and p300

**Figure 5:**
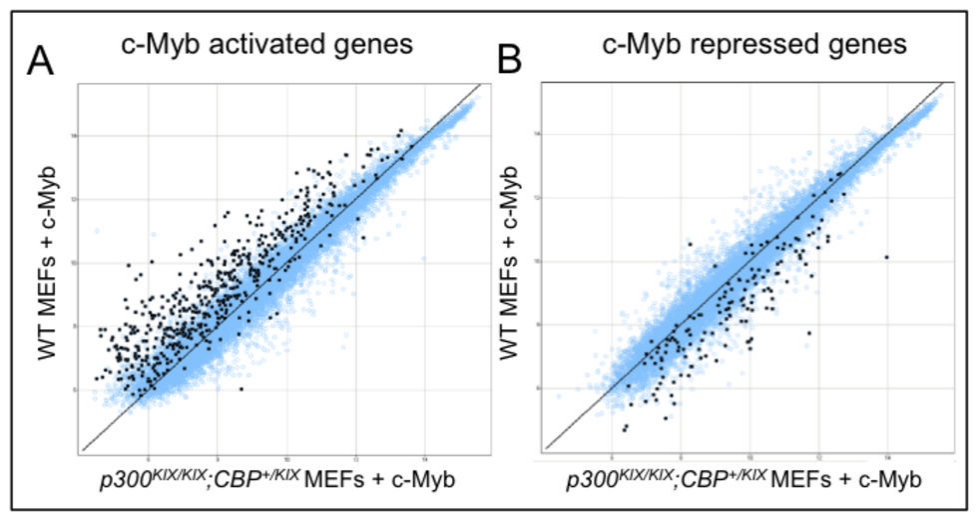
Mutation of the KIX domains of CBP and p300 affects expression of both c-Myb activated and repressed genes. (A,B) Affymetrix gene array of wild type (WT) and p300^*KIX/KIX*^;*CBP* ^+/KIX^ primary mouse embryonic fibroblasts (MEFs) transduced with MSCV c-Myb IRES GFP (c-Myb) retrovirus. Data shown represents average of two biological replicates for each genotype. (A) probe sets shown were scored all present in both WT + c-Myb MEFs. 509 probe sets (marked in black) were induced at least twofold by c-Myb expression in both WT MEFs. (B) probe sets shown were scored all present in WT and p300^*KIX/KIX*^;*CBP* ^+/KIX^ + MSCV IRES GFP control retrovirus MEFs. 148 probe sets (marked in black) were repressed at least 50% by c-Myb expression in both WT MEFs. Axes scales are log_2_.

So far we have shown that KIX and c-Myb mutant mouse phenotypes overlap, and that *p300* ^*KIX*^ *, CBP* ^*KIX*^, and *c-Myb* null mutant alleles can synergize. We next investigated whether c-Myb-dependent gene expression requires the KIX domains of CBP and p300. For our initial studies we chose mouse embryonic fibroblasts (MEFs), over hematopoietic cells for three reasons: 1) sufficient numbers of phenotypically uniform primary MEFs with a *p300* ^*KIX/KIX*^;*CBP* ^+/KIX^ genotype can be obtained; 2) *p300* ^*KIX/KIX*^;*CBP* ^+/KIX^ MEFs are phenotypically similar to wild-type cells and do not have broad transcriptional defects (see blue probe sets in Figure 5A and B); and 3) MEFs possess little or no endogenous c-Myb allowing us to control c-Myb expression by retroviral transduction (Figure S2). In this way we could more confidently identify Myb-responsive genes, and eliminate those genes that might depend on KIX via another transcription factor or are altered by developmental defects. Despite the lack of endogenous c-Myb in MEFs, the expression of many genes increased or decreased more than twofold in response to exogenous c-Myb (black probesets in Figure 5), indicating that necessary cofactors for c-Myb activity are present in these cells. Analysis of Affymetrix microarray probe sets induced at least twofold by exogenous c-Myb in wild type MEFs showed that 382 of 509 probe set signals (75%) were at least 1.5 fold higher in wild type + c-Myb MEFs compared with *p300* ^*KIX/KIX*^;*CBP* ^+/KIX^ + c-Myb MEFs (Figure 5A, probe sets in black and Table S1). Interestingly, of probe sets repressed at least 50% by c-Myb expression in wild type MEFs, 91 of 148 (61%) were at least 1.5 fold higher in *p300* ^*KIX/KIX*^;*CBP* ^+/KIX^ + c-Myb MEFs than wild type + c-Myb MEFs (Figure 5B, probe sets in black and Table S1). Of the 446 genes uniquely identified with probe sets induced or repressed at least twofold by exogenous c-Myb in MEFs, 173 (39%) have previously been shown to recruit c-Myb in their vicinity by ChIP-seq [48]. However, only 26 of these 173 genes were similarly activated or repressed by c-Myb in both MEFs and the ER-inducible c-Myb expressing mouse myeloid precursor cells used in the ChIP-seq study [48] (Table S1) indicating that c-Myb regulation of target genes is highly dependent on cell type. Nonetheless, these data indicate that the KIX domains of CBP and p300 contribute to both c-Myb-dependent activation and repression of transcription.

**Figure 6:**
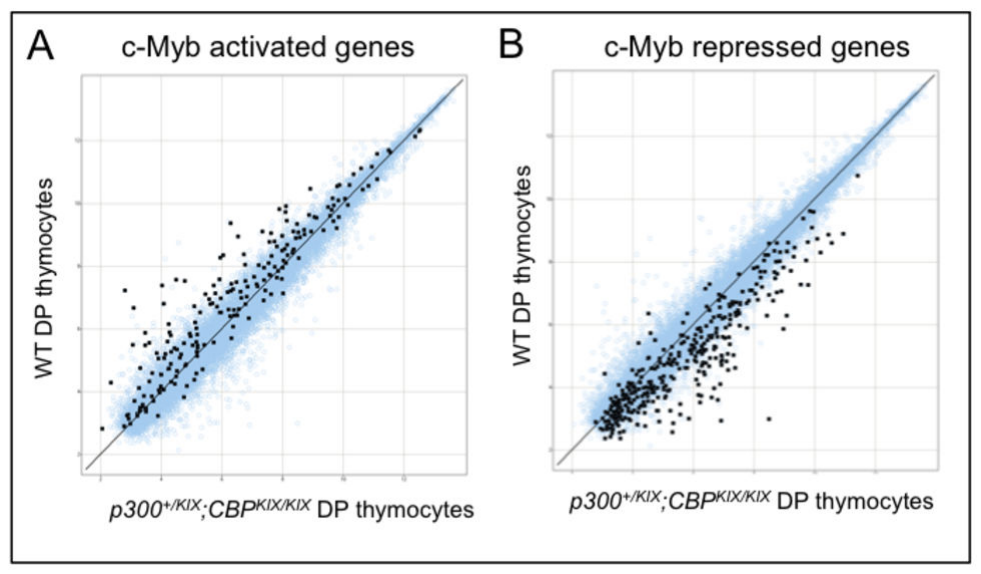
Both c-Myb activated and repressed endogenous genes are sensitive to mutation of the KIX domains of CBP and p300 in thymocytes. (A,B) Affymetrix gene array of wild type (WT) and triple-KIX p300^*+/KIX*^ *; CBP* ^KIX/KIX^ CD4^+^CD8^+^ double positive (DP) thymocytes. Average signal; N=4 mice for each genotype. Probe sets marked in black were identified from data presented in Yuan *et* *al* [70]. and indicate probe sets in which *c-Myb^flox/flox^*;Cd4-Cre;*Tcrα* ^*-/-*^ DP thymocytes were at least twofold higher (A) or at least 50% lower (B) than control cells. For our data sets (black and blue icons), no probes are shown with a signal below 3.4 (twice background). For the Myb-dependent gene set from Yuan et al. used here, probes selected had P<0.05 between Myb null and control DP thymocytes, and none were used where all signals were below 6. Axes scales are log_2_.

Next, we sought to verify that the KIX domain has a similar role in endogenous c-Myb-dependent gene activation and repression using a hematopoietic cell type that normally expresses c-Myb. To do this, we utilized data from a study by Yuan et al. that identified genes regulated by T cell specific inactivation of c-Myb in CD4^+^CD8^+^ double positive (DP) thymocytes (*c-Myb* ^*flox/flox*^;CD4-Cre;*Tcrα* ^*-/-*^ mice compared with *c-Myb* ^*+/flox*^;CD4-Cre;*Tcrα* ^*-/-*^) [70]. We compared these c-Myb activated (Figure 6A, probe sets marked in black, see also Table S2) and repressed (Figure 6B, probe sets marked in black, see also Table S2) gene data sets to Affymetrix microarray analysis of CD4^+^CD8^+^ DP thymocytes FACS purified from adult wild type (WT) and triple-KIX *p300* ^*+/KIX*^;*CBP* ^KIX/KIX^ mice. In general, DP thymocyte gene expression appeared similar between WT and triple-KIX *p300* ^*+/KIX*^;*CBP* ^KIX/KIX^ mice (see blue probe sets in Figure 6A and B); however, when we looked specifically at c-Myb-dependent gene expression (black probe sets in Figure 6A, B and Table S2), the result resembled the situation we observed in c-Myb expressing MEFs (Figure 5). Most c-Myb activated genes defined in c-Myb null DP thymocytes decreased in triple-KIX *p300* ^*+/KIX*^;*CBP* ^KIX/KIX^ DP thymocytes (Figure 6A, black probe sets, Table S2), while most c-Myb repressed genes increased (Figure 6B, black probe sets, Table S2), demonstrating that both activation and repression of many c-Myb-dependent genes require the KIX domain. 

### Both c-Myb activated and repressed gene sets are significantly enriched among genes differentially expressed between triple-KIX *p300* ^*+/KIX*^;*CBP* ^KIX/KIX^ and wild type CD4^+^CD8^+^ double positive thymocytes

**Figure 7:**
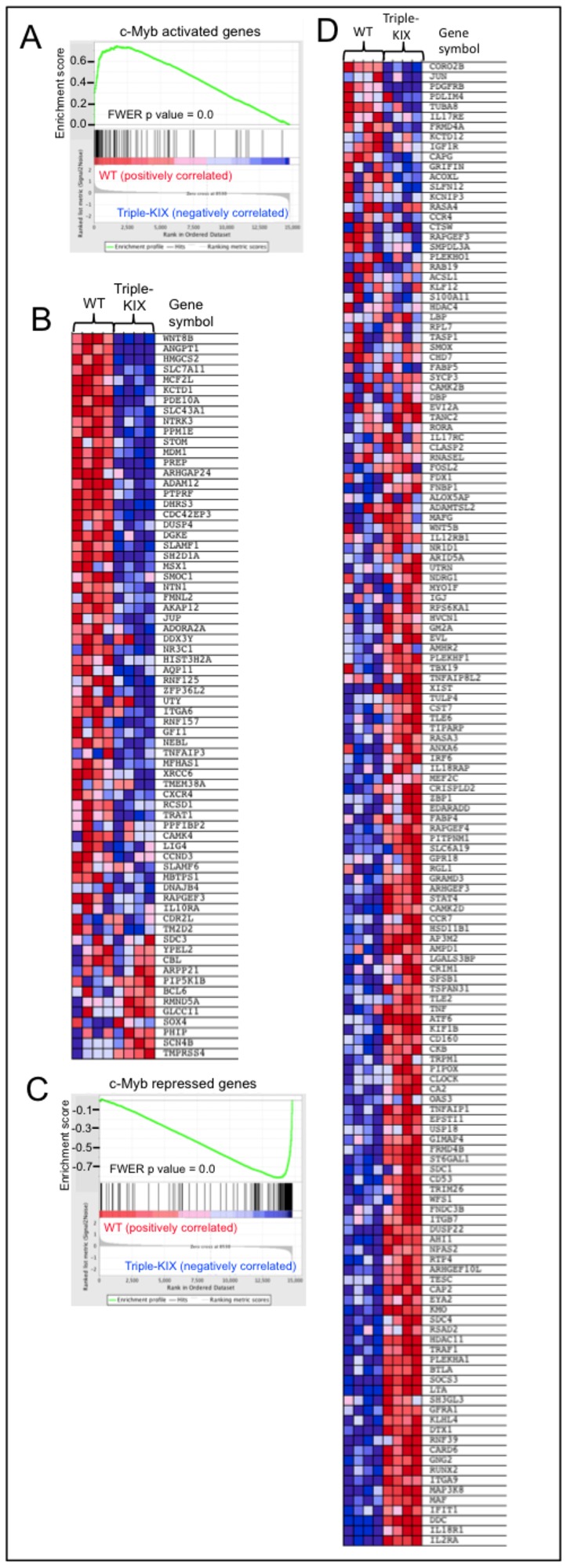
KIX mutation-sensitive genes in DP thymocytes are enriched for genes that are also activated and repressed in c-Myb null DP thymocytes. Gene set enrichment analysis (GSEA [75]) using Affymetrix gene expression data from wild type (WT) and p300^*+/KIX*^; *CBP* ^KIX/KIX^ (triple-KIX) CD4^+^CD8^+^ double positive (DP) thymocytes found significant (FDR q = 0.0, FWER p = 0.0) for enrichment of c-Myb activated (A,B) and repressed (C,D) genes. The two GSEA gene sets shown here were derived from gene expression data for *c*-*Myb* ^*+/flox*^; Cd4-Cre; Tcrα^-/-^ (control) and *c*-*Myb* ^*flox/flox*^; Cd4-Cre; Tcrα^-/-^ (c-Myb null) DP thymocytes [70], which includes c-Myb activated (at least two fold higher in control) and repressed (at least 50% lower in control) genes. In enrichment plots (A, C), genes are ranked by signal/noise ratio according to their differential expression between WT and Triple-KIX DP thymocytes. Genes in the c-Myb gene sets are marked with vertical bars, and the enrichment score is shown in green. Relative expression of the c-Myb activated (B) and repressed (D) gene sets in WT and Triple-KIX DP thymocytes (N=4 mice each) are shown in order of their signal/noise ratio rank.

To assess the statistical significance of this correlation, we included the c-Myb-dependent gene set generated from the c-Myb null CD4^+^CD8^+^ double positive (DP) thymocyte Affymetrix data set of Yuan et al. in a Gene Set Enrichment Analysis (GSEA) of our wild type and triple-KIX *p300* ^*+/KIX*^;*CBP* ^KIX/KIX^ DP thymocyte Affymetrix data [70,75]. GSEA is a computational method used to measure the coordinate regulation of sets of genes defined by biological criteria (e.g. activated or repressed in response to a particular stimulus, dependent on a specific transcription factor or expressed in a certain cell type) even if the changes to individual genes are small [75,76]. Amongst a diverse group of 79 other gene sets we analyzed by GSEA, the two c-Myb-dependent gene sets derived from the data of Yuan et al. were significantly correlated with the KIX mutant data set [Figure 7A-D, false discovery rate (FDR) q = 0.0, family-wise error rate (FWER) p = 0.0] and followed the expected trend (i.e. wild type DP thymocytes expressed c-Myb activated genes more highly than *p300* ^*+/KIX*^;*CBP* ^KIX/KIX^ , and c-Myb repressed genes were expressed lower in wild type than *p300* ^*+/KIX*^;*CBP* ^KIX/KIX^ DP thymocytes). Of the 283 genes uniquely identified with probe sets induced or repressed at least twofold by c-Myb in Yuan et al., 106 (38%) were shown to recruit c-Myb in their vicinity by ChIP-seq using ER-inducible c-Myb expressing myeloid precursor cells [48]. However, for only 20 of these 106 genes was the direction of regulation by c-Myb the same in both DP thymocytes and the myeloid cells used in the ChIP-seq experiment [48,70] (Table S2). While the agreement in DP thymocyte gene expression between KIX dependence in our study and c-Myb gene dependence in the study of Yuan et al. indicates that an intact KIX domain is required for much of c-Myb dependent gene expression, it appears that c-Myb target genes differ substantially between cell types.

### Triple-KIX *p300* ^*+/KIX*^;*CBP* ^KIX/KIX^ and triple-het *p300* ^*+/KIX*^;*CBP* ^*+/KIX*^;*c-Myb* ^*+/-*^ mice recapitulate the abnormal CD4^+^CD8^+^CD25^+^ thymic population associated with loss of c-Myb

**Figure 8:**
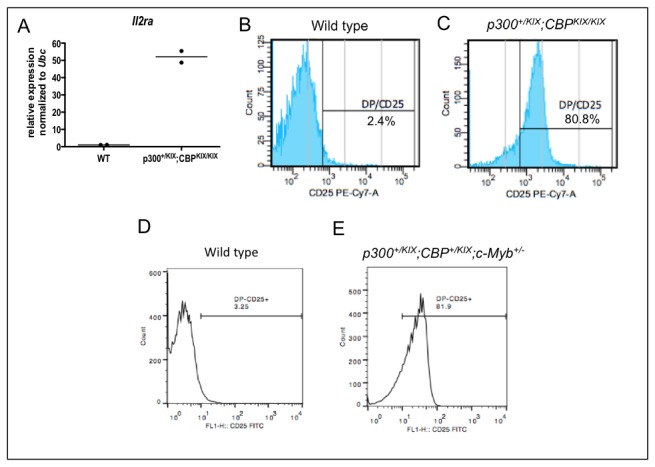
Il2ra (CD25) is abnormally expressed on CD4^+^CD8^+^ thymocytes from mice with KIX and c-Myb complex insufficiencies. (A) qRT-PCR for Il2ra mRNA in CD4^+^CD8^+^ double positive (DP) thymocytes from wild type (WT) and triple-KIX p300^*+/KIX*^;*CBP* ^KIX/KIX^ mice. (B-E) CD25 protein expression on DP thymocytes from wild type (B,D), triple-KIX p300^*+/KIX*^;*CBP^KIX/KIX^* (C) and combined haploinsufficient triple-het p300^*+/KIX*^;*CBP^+/KIX^*;c-Myb^+/-^ (E) mice. 5 week old C57BL/6J x 129Sv F1 (B,C) and 8 week old C57BL/6J (D,E) mice.

In the dataset from Yuan et al., one gene repressed by c-Myb in CD4^+^CD8^+^ DP thymocytes is *Il2ra*, which encodes CD25, the alpha chain of the IL2 receptor (Figure 7D) [70]. We found that triple-KIX *p300* ^*+/KIX*^;*CBP* ^KIX/KIX^ CD4^+^CD8^+^ DP thymocytes upregulated *Il2ra* mRNA (Figure 7D and Figure 8A), showing that intact CBP and p300 KIX domains, like c-Myb, repress *Il2ra* expression. Interestingly, Lieu et al. reported previously that T cell-specific deletion of c-Myb using either Lck-Cre or CD4-Cre produced an abnormal population of CD4^+^CD8^+^ DP thymocytes that were also CD25^+^ [77]. We examined CD25 (IL2RA) protein expression in CD4^+^CD8^+^ DP thymocytes from *p300* ^*+/KIX*^;*CBP* ^KIX/KIX^ mice and found that they had more CD4^+^CD8^+^CD25^+^ thymocytes (79.4% ± 7.5%, mean ± s.d., Figure 8C) than wild type (2.5% ± 0.7%, Figure 8B) or intermediate genotype age matched controls (*p300* ^*+/KIX*^, *CBP* ^KIX/KIX^ and *p300* ^*+/KIX*^;*CBP* ^+/KIX^ mice; 6.0% ± 1.1%, 13.0% ± 2.5% and 28.7% ± 5.0% respectively, Figure S3A-E). Likewise, we observed the same abnormal CD4^+^CD8^+^CD25^+^ thymic population in triple-het *p300* ^*+/KIX*^;*CBP* ^*+/KIX*^;*c-Myb* ^*+/-*^ mice (75.0% ± 9.8%, Figure 8E), which was not present in wild type (3.4% ± 1.8%, Figure 8D) or intermediate genotype age matched controls (Figure S3D,F and G). 

Together, the data suggests that c-Myb and KIX mutations, individually or when combined, result in similar phenotypes and gene expression patterns when considering data from the same cell type (i.e. DP thymocytes). This further supports the idea that c-Myb and CBP/p300 KIX genetically interact in a common pathway to affect many c-Myb-dependent genes and biological processes. Zhao et al. have shown by chromatin immunoprecipitation that p300 is directly recruited to c-Myb activated and repressed target genes [48], but whether the genes affected in this study are also direct targets of c-Myb and CBP/p300 KIX remains a question for future studies. 

## Discussion

### Multiple blood cell lineages and both c-Myb-dependent gene activation and repression require the genetic interaction between c-Myb and the KIX domains of CBP and p300

We show here for the first time that the KIX domain of CBP contributes to the normal production of multiple blood cell lineages, including red cells, B and T cells and megakaryocytes/platelets (Figures 2 and 3). We also found that genetic interaction between *c-Myb* and both *CBP* and *p300* controls megakaryocyte/platelet, B and T cell and likely red cell production (Figure 4). The genetics are consistent with what is known of the physical and functional interaction between c-Myb and the KIX domain, which surprisingly influences both gene activation and repression (Figure 5-8) [20,56,66]. Although none of the KIX mutant genotypes examined displayed myeloid defects, this may be consistent with findings that certain myeloid cells are c-Myb-independent [74,78]. Together, our findings suggest that the c-Myb interaction with the KIX domain of CBP, and especially p300, is a major pathway contributing to the other hematopoietic lineages. 

### The KIX domains of both CBP and p300 participate in the normal formation of multiple blood cell lineages

In our original study, the lack of a blood phenotype in *CBP* ^KIX/KIX^ mice compared with that seen in *p300* ^*KIX/KIX*^ mice surprised us [56]. We hypothesized that this difference was due to the relative amounts of CBP and p300 protein in a critical cell type rather than functional differences between CBP and p300 [56]. The findings of this study support this hypothesis. Triple-KIX *p300* ^*+/KIX*^;*CBP* ^KIX/KIX^ mice display multiple blood lineage defects, while *p300* ^*+/KIX*^ and *CBP* ^KIX/KIX^ mice have normal blood (Figure 3); therefore, in this context, mutation of both CBP and p300 KIX domains is necessary to produce a blood phenotype. Our results agree with the finding that in the absence of endogenous p300 protein, CBP produced from a *CBP* cDNA driven by the *p300* promoter can rescue hematopoiesis [65]. In this regard, p300 KIX is more important than CBP KIX for hematopoiesis in mice, but in other organisms CBP might predominate and it may prove fruitful to look for CBP mutations in human and horse hematopoietic syndromes with phenotypic similarities to the *p300* ^*KIX/KIX*^ mice [56,79,80].

### Multiple blood cell lineages require the genetic interaction between c-Myb and the KIX domains of CBP and p300

Several studies report phenotypic similarities between mice with mutations in the p300 and c-Myb domains through which they physically interact [25,56,81]. The complex haploinsufficiency analysis we performed previously [56] and here, tests for epistasis (gene interaction), which is a method to examine whether c-Myb and KIX might interact *in vivo*. Indeed, triple-het *p300* ^*+/KIX*^;*CBP* ^*+/KIX*^;*c-Myb* ^*+/-*^ mice showed evidence of epistasis, supporting the c-Myb:KIX common pathway hypothesis, most clearly for the lymphoid and megakaryocyte lineages, but also for the tightly-regulated red cell population (Figure 4 and Figure S1).

All of the blood cell lineages affected in p300^*KIX/KIX*^ and triple-KIX *p300* ^*+/KIX*^;*CBP* ^KIX/KIX^ mice show evidence of epistasis in triple-het *p300* ^*+/KIX*^;*CBP* ^*+/KIX*^;*c-Myb* ^*+/-*^ mice compared to intermediate genotype controls (Figure 4). This suggests that all blood cell defects caused by mutation of the KIX domain (anemia, increased platelet count and deficiency of peripheral B and T cells) could be explained by decreased physical interaction of the KIX domain with c-Myb. It further suggests that these lineages are not greatly influenced by effects on other KIX-binding factors like CREB [59].

It remains unclear what role the KIX domains of CBP and p300 play in the c-Myb-dependent production of myeloid cells (Figure 4F,G and H). Previous studies have shown that the total absence of c-Myb does not allow the development of any adult blood cell lineages [1,82]; however, partial reductions in c-Myb protein level or diminutions of the KIX:c-Myb interaction affect blood cell lineages nonuniformly. Low c-Myb protein expression in mice (5-10% of normal) decimates B and T cell numbers and produces fetal liver cells that form few or no red cell or granulocytic myeloid (including neutrophil) lineage colonies, but generate more monocytic myeloid and megakaryocyte lineage colonies than wild type controls (Table 1) [83]. Similarly, mice homozygous for a single amino acid substitution (M303V) in the KIX-binding domain of c-Myb, which results in decreased c-Myb transactivation potential, display decreased lymphocyte counts and hematocrit, elevated numbers of platelets, but normal monocyte and neutrophil numbers (Table 1) [25]. These data suggest a hierarchy: partial c-Myb function impairs B, T and red cell production the most, sometimes spares myeloid cell types, and favors the megakaryocyte/platelet lineage. Indeed, none of the combinations of KIX and c-Myb mutant alleles utilized in this study (*p300* ^*KIX/KIX*^, triple-KIX *p300* ^*+/KIX*^;*CBP* ^KIX/KIX^ or triple-het *p300* ^*+/KIX*^;*CBP* ^*+/KIX*^;*c-Myb* ^*+/-*^) displayed a deficit in the myeloid lineage cells (neutrophils and monocyte/macrophages) (Table 1). It is unclear whether normal production of the myeloid lineages requires less wild type CBP/p300 protein than other blood cell lineages (both triple-KIX *p300* ^*+/KIX*^;*CBP* ^KIX/KIX^ and triple-het *p300* ^*+/KIX*^;*CBP* ^*+/KIX*^;*c-Myb* ^*+/-*^ mice have some wild type CBP and/or p300 protein), if c-Myb interacts with CBP/p300 via domains other than KIX or utilizes other cofactors, or if the remaining myeloid cells are c-Myb-independent [74,78]. 

### The KIX domains of CBP and p300 contribute to both c-Myb-dependent activation and repression of transcription

Our data from both c-Myb-expressing mouse embryonic fibroblasts (Figure 5) and CD4^+^CD8^+^ double positive thymocytes (Figure 6) show that much of c-Myb-dependent gene expression depends on the KIX domain. Since in both of our model systems one wild type allele of either *CBP* or *p300* remains, we could not determine whether the unaffected minority of c-Myb-dependent genes are truly KIX-independent or if they receive sufficient coactivation from the remaining wild type CBP or p300. 

Our observation that c-Myb repressed many genes in a KIX-dependent manner (Figures 5B and 6B) was initially unexpected. However, Zhao et al. recently showed using an estrogen inducible ER-c-Myb fusion expressing myeloid cell line that c-Myb and p300 are directly recruited to both c-Myb activated and repressed target genes [48]. They also compared bone marrow cells transduced with a retrovirus expressing wild type or L302A c-Myb (the L302A mutation ablates the interaction of c-Myb with the KIX domain [66]) and showed that most direct c-Myb activated (15 of 19) and repressed (29 of 34) genes tested were sensitive to L302A [48]. Analysis of granuloid/myeloid progenitor (GMP) cells, which express endogenous c-Myb, from wild type and *Myb* ^*E308G/E308G*^ mutant mice (the E308G mutation decreases c-Myb interaction with the KIX domain) [84] showed a similar mutation sensitivity for both c-Myb activated and repressed gene expression [48]. Our study did not address whether the c-Myb- and CBP/p300 KIX-dependent genes we observed were direct or indirect targets of these factors. However, our expression data nicely complement the study of Zhao et al. and demonstrate a clear role for the KIX domain in both c-Myb activated and repressed gene sets (Figures 5-7). 

### c-Myb may either activate or repress genes in a context dependent manner

**Figure 9:**
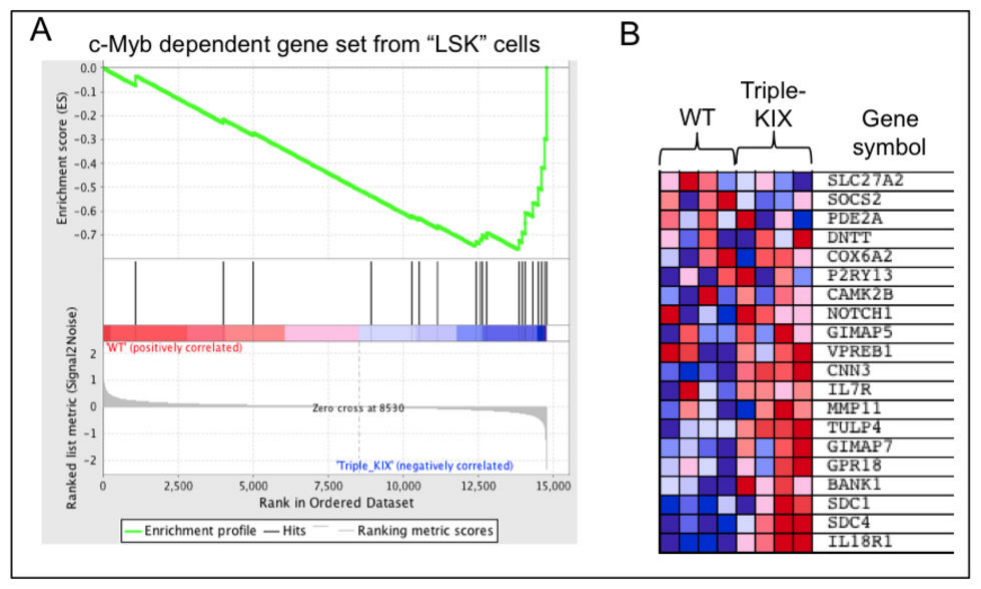
c-Myb dependent gene set identified in c-Myb null Lin^-^ Sca-1^+^ c-kit^+^ (LSK) cells is enriched in KIX mutant DP thymocytes, but displays a largely reversed expression profile from expected. Gene set enrichment analysis (GSEA) using Affymetrix gene expression data from wild type (WT) and p300^*+/KIX*^; *CBP* ^KIX/KIX^ (Tri-KIX) CD4^+^CD8^+^ double positive (DP) thymocytes found significant (FDR q = 0.00079, FWER p = 0.0030) enrichment of a set of genes dependent on c-Myb in LSK cells as defined by [71]. (A) In the enrichment plot, genes are ranked by signal/noise ratio according to their differential expression between WT and Tri-KIX DP thymocytes. Genes in the LSK gene set are marked with vertical bars, and the enrichment score is shown in green. (B) Relative expression of the LSK gene set in WT and Tri-KIX DP thymocytes (N=4 mice each) are shown in order of their signal/noise ratio rank.

In comparing c-Myb-dependent genes from different studies, we were struck by how little overlap exists between the datasets [48,71,85-88]. This has been a challenge for those attempting to define c-Myb target genes and it is unclear how much can be explained by differences in cell types used and study design. Interestingly, recent attention to c-Myb repressed genes may offer a clue in this mystery. During GSEA analysis, we found that a gene expression dataset derived from analysis of wild type and *Myb* ^Plt4/Plt4^ Lin^-^ Sca-1^+^ c-kit^+^ (LSK) hematopoietic precursor cells [71] was highly enriched in our analysis of wild type and *p300* ^*+/KIX*^;*CBP* ^KIX/KIX^ CD4^+^CD8^+^ double positive (DP) thymocytes (Figure 9, FDR q = 0.00079, FWER p = 0.0030). Although the Plt4 mutation (V384D) in Myb does not disrupt the interaction with CBP KIX or full-length p300 [89], it produces a similar phenotype to that seen in *p300* ^*KIX/KIX*^ mice [90]. Surprisingly, the GSEA correlation we observed is largely reversed; most genes that are repressed in *Myb* ^*Plt4/Plt4*^ LSK cells showed increased expression in *p300* ^*+/KIX*^;*CBP* ^KIX/KIX^ DP thymocytes compared to wild type (Figure 9). These data suggest that c-Myb in collaboration with CBP and p300 can either repress or activate the same genes based on as yet unknown cellular cues (e.g. LSK cells vs. DP thymocytes). 

In all, our data suggest that while the KIX domain of p300 is more critical for murine hematopoiesis, the KIX domain of CBP also participates. We have shown that the functional interaction between c-Myb and the KIX domains of CBP and p300 impacts all of the blood cell lineages dysregulated by CBP/p300 KIX domain mutations; however, it remains unknown whether c-Myb-dependent myeloid lineage production also requires the c-Myb:KIX interaction. Lastly, while CBP and p300 are primarily known for their roles as coactivators, mutation of the KIX domain impairs both the repression and the activation of c-Myb-dependent genes. These findings provide mechanistic insight into how c-Myb, p300, and CBP influence hematopoiesis, and establish the importance of the interaction between CBP/p300 KIX and c-Myb for multiple blood cell lineages. 

## Supporting Information

Figure S1 **Combined KIX and c-Myb insufficiency produces a genetic interaction that affects reticulocyte number.** Peripheral blood counts from 3-12 month old C57BL6x129Sv (F1) background mice. Counts from automated Hemavet complete blood count. Asterisks indicate significant p value by pairwise Tukey post test following one way ANOVA (* p<0.05, ** p<0.01, *** p<0.001). p300^+/KIX^ and p300^+/KIX^;c-Myb^+/-^ data were left out of ANOVA and Tukey post test analyses because these genotypes were represented by a single mouse in this experiment. ANOVA p=0.0002.
(TIF)

Figure S2 **Primary mouse embryonic fibroblasts (MEFs) have little or no endogenous c-Myb.** Western blot of whole cell extracts from wild type and *CBP^+/KIX^*;*p300* ^*KIX/KIX*^ MEFs transduced with c-Myb or control retrovirus (all retroviruses used express Green Fluorescent Protein (GFP) from an internal ribosomal entry site (IRES), c-Myb L302A is a c-Myb mutant that is not reported in this study). c-Myb was detected with clone 1-1 monoclonal antibody from Millipore.
(TIF)

Figure S3 **CD25 is abnormally expressed on CD4^+^CD8^+^ double positive thymocytes from triple-KIX *p300*^*+/KIX*^;*CBP*^KIX/KIX^ mice, but intermediate KIX mutant genotypes as well as c-Myb^+/-^ mice are much less affected.** CD25 expression on CD4^+^CD8^+^ double positive (DP) thymocytes from 5 week old C57Bl/6Jx129Sv F1 (A-E) and 4 week old C57Bl/6J (F,G) mice.
(TIF)

Table S1 **Affymetrix microarray probe sets from Figure 5 (from microarray data set deposited with ArrayExpress (E-MTAB-1973)) showing the expression in wild type (WT) and *p300*^*KIX/KIX*^; *CBP*^+/KIX^ primary mouse embryonic fibroblasts (MEFs) transduced with MSCV c-Myb IRES GFP (c-Myb) retrovirus of genes that were induced or repressed at least twofold by exogenous c-Myb in wild type MEFs.**
(XLSX)

Table S2 **Affymetrix microarray probe sets from Figure 6 (from microarray data set deposited with ArrayExpress (E-MTAB-1974)) showing the expression in wild type (WT) and triple-KIX *p300*^*+/KIX*^** ;***CBP*^KIX/KIX^ CD4^+^CD8^+^ double positive (DP) thymocytes of genes that were induced or repressed at least twofold by c-Myb KO in a study by Yuan et al. that identified genes regulated by T cell specific inactivation of c-Myb in CD4^+^CD8^+^ double positive (DP) thymocytes**.
(XLSX)
