# Supplementary figures and images for: Genetic Interaction between Mutations in c-Myb and the KIX Domains of CBP and p300 Affects Multiple Blood Cell Lineages and Influences Both Gene Activation and Repression
Source: PLoS One. 2013 Dec 10;8(12):e82684. doi: 10.1371/journal.pone.0082684 (PMC3858336; doi:10.1371/journal.pone.0082684)

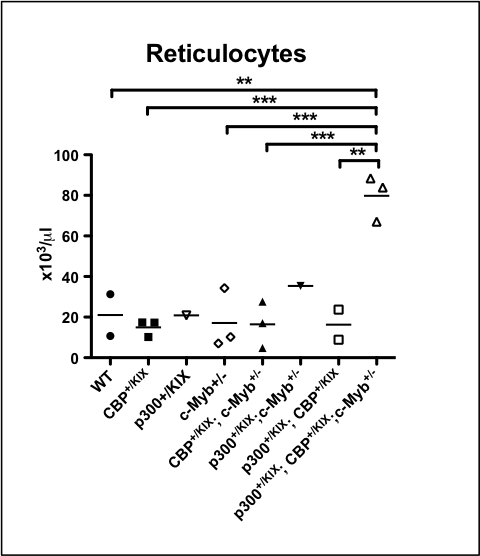

Supplement: Figure S1 — Combined KIX and c-Myb insufficiency produces a genetic interaction that affects reticulocyte number. Peripheral blood counts from 3-12 month old C57BL6x129Sv (F1) background mice. Counts from automated Hemavet complete blood count. Asterisks indicate significant p value by pairwise Tukey post test following one way ANOVA (* p<0.05, ** p<0.01, *** p<0.001). p300+/KIX and p300+/KIX;c-Myb+/- data were left out of ANOVA and Tukey post test analyses because these genotypes were represented by a single mouse in this experiment. ANOVA p=0.0002. (TIF) [file pone.0082684.s001.tif]

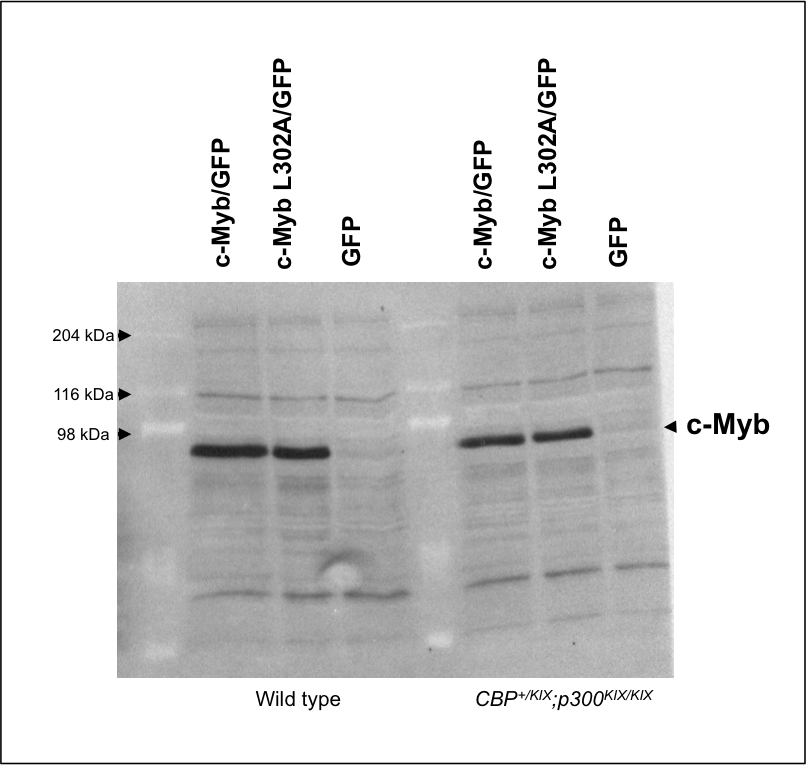

Supplement: Figure S2 — Primary mouse embryonic fibroblasts (MEFs) have little or no endogenous c-Myb. Western blot of whole cell extracts from wild type and CBP+/KIX;p300 KIX/KIX MEFs transduced with c-Myb or control retrovirus (all retroviruses used express Green Fluorescent Protein (GFP) from an internal ribosomal entry site (IRES), c-Myb L302A is a c-Myb mutant that is not reported in this study). c-Myb was detected with clone 1-1 monoclonal antibody from Millipore. (TIF) [file pone.0082684.s002.tif]

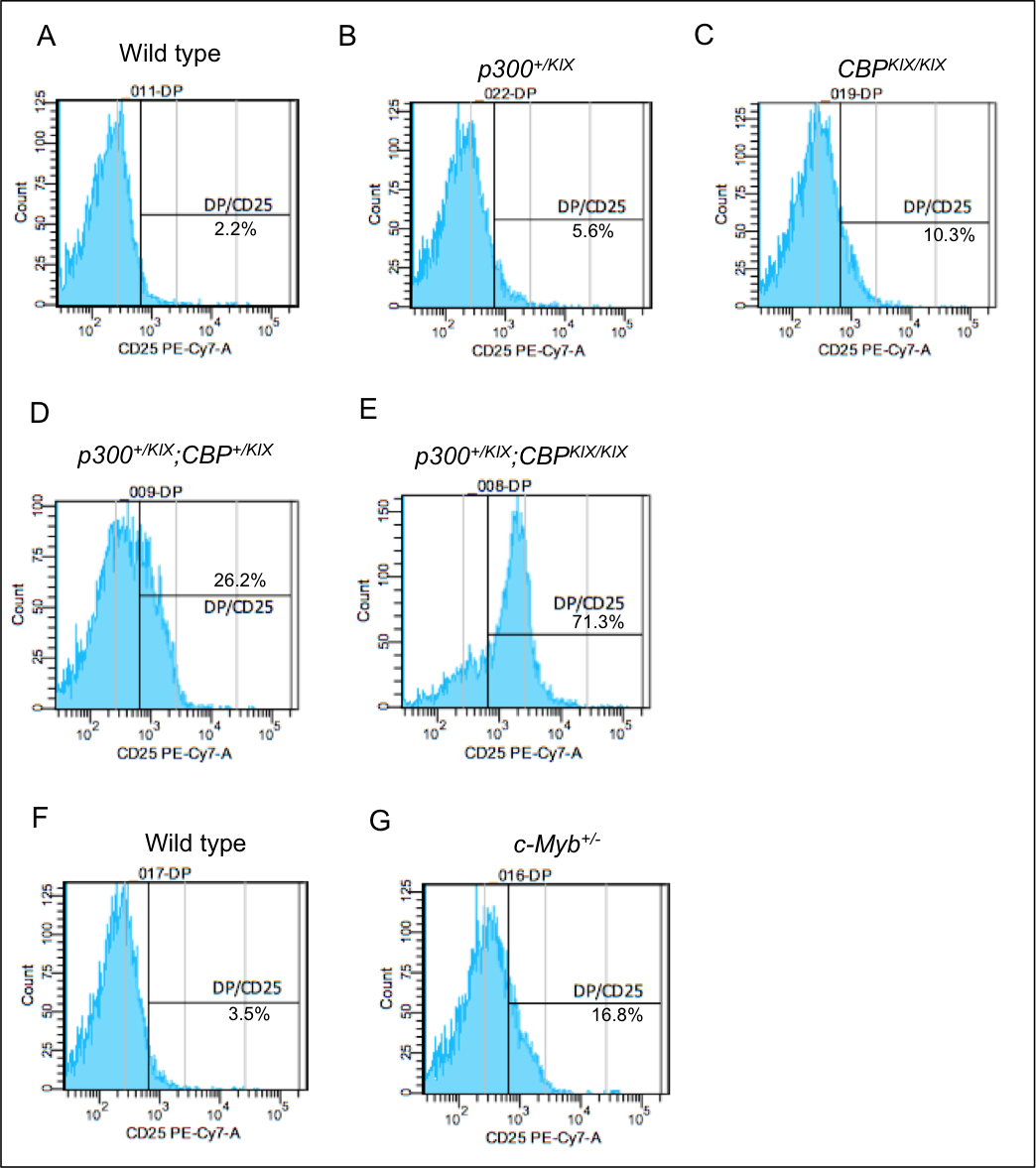

Supplement: Figure S3 — CD25 is abnormally expressed on CD4+CD8+ double positive thymocytes from triple-KIX p300+/KIX;CBPKIX/KIX mice, but intermediate KIX mutant genotypes as well as c-Myb+/- mice are much less affected. CD25 expression on CD4+CD8+ double positive (DP) thymocytes from 5 week old C57Bl/6Jx129Sv F1 (A-E) and 4 week old C57Bl/6J (F,G) mice. (TIF) [file pone.0082684.s003.tif]
